# Advances in ovarian cancer therapy

**DOI:** 10.1007/s00280-017-3501-8

**Published:** 2017-12-16

**Authors:** Alexander J. Cortez, Patrycja Tudrej, Katarzyna A. Kujawa, Katarzyna M. Lisowska

**Affiliations:** Maria Skłodowska-Curie Institute - Oncology Center, Gliwice Branch, Wybrzeże Armii Krajowej 15, Gliwice, 44-100 Poland

**Keywords:** Ovarian cancer, Biological drugs, Targeted therapy, Clinical trials

## Abstract

Epithelial ovarian cancer is typically diagnosed at an advanced stage. Current state-of-the-art surgery and chemotherapy result in the high incidence of complete remissions; however, the recurrence rate is also high. For most patients, the disease eventually becomes a continuum of symptom-free periods and recurrence episodes. Different targeted treatment approaches and biological drugs, currently under development, bring the promise of turning ovarian cancer into a manageable chronic disease. In this review, we discuss the current standard in the therapy for ovarian cancer, major recent studies on the new variants of conventional therapies, and new therapeutic approaches, recently approved and/or in clinical trials. The latter include anti-angiogenic therapies, polyADP-ribose polymerase (PARP) inhibitors, inhibitors of growth factor signaling, or folate receptor inhibitors, as well as several immunotherapeutic approaches. We also discuss cost-effectiveness of some novel therapies and the issue of better selection of patients for personalized treatment.

## Introduction

Ovarian cancer is the second most common and the most lethal gynecologic malignancy in the western world. So far, there is lack of methods recommended for screening and early diagnostics of this disease. As a consequence, and also due to the absence of early warning symptoms, about 70% of cases is diagnosed at an advanced stage and have bad prognosis. Late-stage ovarian cancer is incurable in the majority of cases, but recently it tends to become a kind of chronic disease. This is mostly due to the progress in surgical technology and contemporary regimes of systemic treatment, as well as some new drugs entering the clinic.

Currently, there are also many new drugs under development and tested in the ongoing clinical trials aimed to evaluate their efficacy in the treatment of ovarian cancer. New drugs are mostly directed against molecular targets and pathways that are indispensable for cancer cells proliferation, tumor growth and escape from immune surveillance and death signals. These are, e.g., anti-angiogenic factors, inhibitors of growth factor signaling, polyADP-ribose polymerase (PARP) inhibitors, or folate receptor inhibitors. In addition, there are many immunotherapeutic approaches tested. So far, these new agents and therapeutic approaches were not shown to cure ovarian cancer, but they may improve therapy and lead to the delay of recurrence or stabilization of the disease.

However, the landscape of ovarian cancer treatment is complicated by heterogeneity of these tumors. Different histological types of epithelial ovarian cancer have distinct cellular origin, diverse mutational spectrum, and thus, different prognosis (rev. in: [1, 2]). Even within one histological type, distinct molecular subtypes with different prognoses can be found (see e.g.: [3, 4]). To address these issues there is a need to better characterize these differences, find reliable biomarkers and develop appropriate targeted therapies. Even though many studies are aimed at biomarker discovery, and many putative biomarkers are published, very few are finally entering the clinics [5].

In this review, we discuss current standard in the therapy for ovarian cancer and new therapeutic approaches, and their present status.

## Standard treatment for ovarian cancer

The standard treatment for ovarian cancer is maximal cytoreductive surgical debulking followed by the platinum-based chemotherapy. Confirmation of the diagnosis, as well as staging of the disease is performed during surgery.

In any case, efforts should be made to define the histological type of the tumor, including grading [6]. High-grade/low-grade scale is currently used, except for endometrioid ovarian cancer where a three-grade scale is used (G1, G2 or G3) [7]. Staging assessment in surgical-pathologic degrees should be done according to current FIGO recommendations [8].

According to the Gynaecologic Oncology group (GOG), optimal cytoreduction was previously defined as residual tumor nodules each measuring 1 cm or less in maximum diameter. However, large multivariate analysis showed improved progression-free and overall survival for group of patients with complete resection compared with groups with the so-called optimal (between 0.1 and 1 cm) and suboptimal cytoreduction (*p* < 0.0001) [9]. Thus, according to the 2017 ESGO ovarian cancer surgery guidelines, the aim of the frontline surgery is to achieve complete resection of macroscopic residuals of the disease (complete cytoreduction) [10].

After surgery, patients are treated with the intravenous platinum/taxane regimes, every 21 days, for six cycles (first-line chemotherapy). In patients with stage IA/IB and with G1/G2 tumors, the chemotherapy can be omitted [6].

In advanced stages (III/IV), complete cytoreduction is often not possible. The most common reason is the seizure of small bowel mesentery and the lesions in the liver hilum. Patients with inoperable lesions or due to poor performance status are first treated with induction (neoadjuvant) chemotherapy. After three cycles of the chemotherapy, if there is a response to the treatment, the interval debulking surgery (IDS) can be performed, then chemotherapy is continued, up to six cycles [6].

Treatment outcome is assessed after the completion of first-line chemotherapy. Evaluation of response to the treatment is done based on imaging results and according to RECIST 1.1 criteria (Response Evaluation Criteria In Solid Tumors) [11]. The majority of patients respond well to the first-line chemotherapy, achieving complete response (CR), however, many will develop recurrence. For patients with residual disease < 1 cm, the risk for recurrence is estimated at 60–70%; for women with large-volume residual disease, the risk is estimated at 80–85% [12]. Therefore, patients with CR should be subjected to periodic controls. Increasing level of CA125 can be an early symptom of recurrence, however, if not accompanied by clinical symptoms, it is not recommended to implement second-line treatment. Deferral of treatment, until clinical symptoms occur, does not worsen the survival [13]. There is consensus that patients with recurrent disease on the basis of CA125 alone, are eliglible for clinical trials [14].

### New approaches to the first-line treatment

Phase III clinical trials indicate that the incorporation of targeted anti-angiogenic treatment with bevacizumab and weekly dose-dense paclitaxel into first-line management of ovarian cancer can improve survival. Thus, both of these approaches can be considered new standards-of-care. However, they have markedly different economic implications and place distinct burdens on patients (higher toxicity and intensity of therapy).

In 2011, based on data from Gynecologic Oncology Group protocol 0218 (GOG0218/NCT00262847) and International Collaboration for Ovarian Neoplasia 7 (ICON7/ NCT00483782) trials, bevacizumab has gained the European Medicines Agency (EMA) approval for the first-line treatment together with standard chemotherapy (carboplatin and paclitaxel) in women with advanced epithelial ovarian cancer, fallopian tube cancer or primary peritoneal cancer (OFPC) [15]. However, FDA has not approved bevacizumab for the first-line treatment, so far (decision is expected by June 2018).

The results of the Japanese GOG 3016 trial (NCT00226915) suggested that dose-dense weekly paclitaxel plus carboplatin improved survival compared with the conventional regimen. Median progression-free survival (PFS) was longer in the dose-dense treatment group (28.0 months, 95% CI 22.3–35.4) than in the conventional treatment group (17.2 months, 15.7–21.1; HR 0.71; 95% CI 0.58–0.88; *p* = 0.0015). Overall survival at 3 years was higher in the dose-dense regimen group (72.1%) than in the conventional treatment group (65.1%; HR 0.75, 0.57–0.98; *p* = 0.03) [16, 17]. On the contrary, the GOG 0262 trial (NCT01167712) showed that weekly paclitaxel, as compared with conventional regimen, did not prolong PFS among patients with ovarian cancer (14.7 versus 14.0 months; HR = 0.89; 95% CI 0.74–1.06; *p* = 0,18). However, it must be mentioned that 84% of analyzed patients received bevacizumab. Among patients who did not receive bevacizumab, weekly paclitaxel was associated with PFS 3.9 months longer than that observed in the conventional treatment group (14.2 versus 10.3 months; HR = 0.62; 95% CI 0.40–0.95; *p* = 0.03). These results support the benefit of weekly paclitaxel plus carboplatin, but in the absence of bevacizumab administration [18]. International Collaboration for Ovarian Neoplasia 8 trial (ICON8/ NCT01654146) is a randomized, three-arm, phase III study designed to investigate again if weekly chemotherapy is more effective than standard chemotherapy. ICON8B is investigating the combination of dose-dense chemotherapy and bevacizumab in a subgroup of women with high-risk stage III–IV ovarian cancer [19]. No results of these trials are published so far.

There is also ongoing debate whether neoadjuvant chemotherapy and IDS may be superior to the massive primary debulking surgery (PDS) in advanced ovarian cancer. The second approach is related with higher mortality and morbidity while the first one may lead to earlier recurrence and shorter survival. The results of a European Organization for Research and Treatment of Cancer (EORTC) 55971 trial (NCT00003636) suggested that patients with stage IIIC and less extensive metastatic tumors had higher survival with primary surgery, while patients with stage IV disease and large metastatic tumors had better survival with neoadjuvant chemotherapy. For patients who did not meet these criteria, both treatment options led to comparable survival rates [20]. Two new trials: SUNNY (Study of Upfront Surgery versus Neoadjuvant Chemotherapy in Patients With Advanced Ovarian Cancer, NCT02859038) and TRUST (Trial on Radical Upfront Surgery in advanced Ovarian Cancer, NCT02828618) were recently started, aimed to compare the OS after PDS versus IDS following the neoadjuvant chemotherapy in patients with FIGO stage IIIB–IVB OFPC.

A majority of ovarian cancers are chemosensitive and are confined to the surface of the peritoneal cavity for a long time. These features decide that ovarian cancer is a good target for intraperitoneal (IP) chemotherapy. A recent meta-analysis explored the results from nine randomized controlled clinical trials, assessing 2119 women with primary epithelial ovarian cancer, of any FIGO stage, after PDS [21]. Standard intravenous (IV) chemotherapy was compared with chemotherapy that included a component of IP administration. Women were less likely to die if they received an IP component to chemotherapy (8 studies, 2026 women; HR = 0.81; 95% CI 0.72–0.90). IP component chemotherapy prolonged the disease-free interval (5 studies, 1311 women; HR = 0.78; 95% CI 0.70–0.86). There was greater serious toxicity with regard to gastrointestinal effects, pain, fever and infection but less ototoxicity with the IP than the IV route. However, the last IP study, GOG 252, failed to show an advantage of IP over IV administration [22]. Thus, it is still not clear whether IP chemotherapy increases OS and PFS. Additionally, the potential for catheter related complications and toxicity must be considered.

### Treatment of recurrence

Despite the high response rate to primary treatment, majority of patients will develop recurrence [23]. Major option for the treatment of recurrent ovarian cancer is chemotherapy.

An important prognostic factor is the time from the end of the previous treatment (treatment-free interval, TFI). The time to relapse is also used as a determinant of tumor sensitivity to platinum. Tumors are categorized as:

- platinum refractory—when tumor progresses during first-line treatment
- platinum resistant—recurrence within 6 months after completion of first-line treatment
- partially sensitive—recurrence within 6–12 months
- highly sensitive—recurrence after more than 12 months

This classification is commonly used, although it is now generally appreciated that platinum sensitivity is a continuum, rather than related to arbitrary time points, and cannot be accurately determined by progression-free interval (PFI) alone [14].

Selection of second-line chemotherapy protocol is based on tumor sensitivity to platinum derivatives. Patients that have partially- or highly-sensitive tumors can be treated with platinum in combination with other drugs. These patients benefit from multi-drug regimens. Usually carboplatin or cisplatin is used in combination with paclitaxel or pegylated liposomal doxorubicin (PLD) or gemcitabine (with or without bevacizumab). For treatment of partially-sensitive recurrences, when platinum is not an option (anaphylaxy to platinum compounds), PLD with trabectedin can be used [24]. As was shown in OVA-301 phase III study (NCT00113607), the patients with mutation in BRCA gene have longer PFS and OS with this regimen [25]. Trabectedin alone was tested in MITO15 phase II trial (NCT01772979) for the treatment for recurrent ovarian cancer patients presenting BRCA mutation and/or BRCA-ness phenotype (≥ 2 previous responses to platinum). It was concluded that the signature of ‘repeated platinum sensitivity’ identifies patients highly responsive to trabectedin which can be valuable alternative option in patients who present contraindication to receive platinum [26].

The prognosis in patients refractory or resistant to platinum treatment is bad. In this group of patients, no benefit from combination therapy was shown over monotherapy with PLD, topotecan, gemcitabine or paclitaxel. The combination of chemotherapy with bevacizumab significantly prolongs progression-free survival (PFS), however, only patients with good performance status are eligible for this treatment.

In certain cases of recurrent ovarian cancer, resection may be considered. It is eligible for patients who had a complete remission and at least 12 months disease-free period after first-line treatment, and with a likelihood of successful radical surgery [14]. The Arbeitsgemeinschaft Gynaekologische Onkologie (AGO) Group DESKTOP OVAR I trial, based on retrospective analysis, showed three factors being independently associated with complete resection: macroscopically complete resection at first surgery, good performance status, and the absence of ascites greater than 500 ml. These three factors were combined to the “AGO-score” that was deemed positive if all three criteria were fulfilled. Survival analysis showed median OS of 45.2 months in completely debulked patients, as compared with 19.7 months in patients with incomplete resection (HR = 3.71; 95% 2.27–6.05; *p* < 0.0001) [27, 28]. AGO score was verified in a prospective trial—AGO DESKTOP OVAR II (NCT00368420). The rate of complete resection was 76%, although negative score might not exclude the possibility to achieve a complete resection. AGO DESKTOP OVAR III (ENGOT ov20/NCT01166737) is randomized, phase III trial comparing second-line chemotherapy versus secondary cytoreductive surgery followed by chemotherapy, in patients with platinum-sensitive recurrent ovarian cancer with a positive AGO-score. OS data are not mature yet, but median PFS was significantly improved in the experimental arm (14 months without versus 20 months with surgery; HR = 0.66; 95% CI 0.52–0.83; *p* < 0.001), even in those patients were complete cytoreduction was not achieved [29]. In summary, DESKTOP trials showed that it is possible to select patients who might benefit from secondary cytoreductive surgery.

There are several recently completed and ongoing clinical trials designed to evaluate new approaches for treatment for recurrent ovarian cancer, e.g. bevacizumab re-treatment (MITO16MANGO2b/NCT01802749; AGO-OVAR 2.21/NCT01837251), PARPi in combinations with other biological drugs, as chemotherapy free option (ENGOT-OV24/AVANOVA/NCT02354131; NRG004/NCT02446600; AGO-OVAR 2.28/ENGOT-ov28) [30], PARPi-based options as maintenance therapy (SOLO 2/NCT01874353, ICON 9 [31]), and immune checkpoint inhibitors (ATALANTE/NCT02891824; AGO-OVAR 2.29 [30]).

## Hyperthermic intraperitoneal chemotherapy (HIPEC)

Recently, a combination of cytoreductive surgery and hyperthermic intraperitoneal chemotherapy (HIPEC) is increasingly used for the management of peritoneal metastases. This procedure is now accepted as a standard treatment for pseudomyxoma peritonei, peritoneal mesothelioma and the peritoneal metastases from colorectal cancer. At some medical centers, HIPEC is also used for the treatment of patients with ovarian cancer.

In ovarian cancer patients, HIPEC is applied in combination with systemic therapy which starts about three weeks after surgery. Cisplatin (optionally with doxorubicin) and taxanes are used most frequently for HIPEC. Best results are achieved in the treatment of platinum-sensitive tumors, although it is suggested that eligible are patients with late recurrences and after several lines of chemotherapy. This therapy could also apply for the patients with large residual disease after the primary surgery and for those who have inoperable lesions. In the latter case, neoadjuvant chemotherapy is given, and the patients, which will respond, qualify for the cytoreductive surgery combined with HIPEC. Another eligible group could include patients in whom laparoscopy revealed malignancy, instead of apparently benign tumor. HIPEC is not recommended when the disease has disseminated to the distant organs outside peritoneum [32].

HIPEC is criticized due to relatively high morbidity and mortality of the procedure. Major complications include anastomotic leakage, bowel perforation, intraperitoneal hemorrhage and wound dehiscence. Reported morbidity rates range from 0 to 31.3% (Grade 3 and 4 morbidity according to the Clavien–Dindo classification) and mortality rates from 0 to 4.2%. Some authors argue that these numbers are similar to those observed in patients undergoing cytoreductive surgery alone [33].

So far, most of the results concerning HIPEC in ovarian cancer are coming from phase I–II or retrospective studies, e.g., a case series analysis (246 ovarian cancer patients with recurrent intraperitoneal lesions or with persistent lesions after systemic treatment) showed that the median overall survival was 49 months after the maximum cytoreductive surgery and HIPEC [34, 35]. Several randomized studies investigating HIPEC are currently ongoing. A large randomized study (280 patients to be enrolled) conducted by the Netherlands Cancer Institute OVHIPEC (NCT00426257) and another smaller study CHORINE (NCT01628380) are investigating the benefit of HIPEC after IDS for primary ovarian cancer. The largest ongoing randomized study (444 patients to be enrolled) is the French CHIPOR study (NCT01376752), evaluating the efficacy of HIPEC in patients with platinum-sensitive recurrent disease. Two other randomized HIPEC trials (NCT01539785 and NCT01767675) are also enrolling patients with recurrent disease, and another is investigating the role of HIPEC after frontline cytoreductive surgery (NCT01091636). Most interestingly, randomized NCT02124421 trial is comparing the efficacy of cytoreductive surgery with HIPEC, and IV chemotherapy versus cytoreductive surgery and post-operative IP and IV chemotherapy in primary ovarian cancer.

Before the results of these studies will be published, and taking into account toxicity of HIPEC, at present, this technique cannot be recommended in daily practice.

## New therapeutic targets in ovarian cancer therapy

### Angiogenesis inhibitors

Angiogenesis is a tightly controlled dynamic process that occurs primarily in embryo development, during wound healing and in response to ovulation. However, it can be aberrantly activated during many pathological conditions such as cancer, diabetic retinopathy as well as numerous ischaemic, inflammatory, infectious and immune disorders. Among known regulators of angiogenesis are growth factors, matrix metalloproteinases, cytokines, and integrins. A key player in the development of the pathological vascular network of tumor is Vascular Endothelial Growth Factor (VEGF) and its signaling pathway. It was initially expected that blocking VEGF signaling in cancer will inhibit angiogenesis and cause tumor shrinkage, due to the reduced blood supply. However, a variety of preclinical studies supported an alternative hypothesis that anti-angiogenic agents can transiently ‘‘normalize’’ the abnormal structure and function of tumor vasculature to make it more efficient for oxygen and drug delivery [36].

In epithelial ovarian cancer, increased VEGF expression has a prognostic value: it is related with tumor grade, stage of the disease, and patients’ survival. As VEGF receptors are present on the surface of ovarian cancer cells, it seems that VEGF may play a unique role in the development of this malignancy. By increasing vascular permeability within peritoneum, VEGF is also responsible for the formation of ascitic fluid in ovarian cancer patients. Consequently, inhibition of pathological angiogenesis became one of the new therapeutic options widely tested in ovarian cancer treatment; promising results are shown with bevacizumab, cediranib and pazopanib, as well as aflibercept.

### Inhibition of VEGF: bevacizumab

Bevacizumab is a recombinant humanized monoclonal antibody against VEGF. It prevents VEGF from binding to its receptor; it was shown that bevacizumab leads to normalization of tumor vasculature and reduction of the interstitial tumor pressure, improving effectiveness of standard therapy. In 2004, it became the first clinically approved angiogenesis inhibitor in the U.S. (approval for the treatment, in combination with standard chemotherapy, for colon cancer) [37]. In 2011, based on data from GOG0218 and ICON7 trials, bevacizumab has gained European Commission approval for the first-line treatment together with standard chemotherapy in women with advanced OFPC [38]. In 2014, the Food and Drug Administration (FDA) approved bevacizumab, in combination with paclitaxel, topotecan or pegylated liposomal doxorubicin (PLD) for the treatment of patients with platinum-resistant recurrent epithelial ovarian, fallopian tube and primary peritoneal cancer [37]. Phase III clinical trials investigating bevacizumab in ovarian cancer, those completed and still ongoing are widely reviewed in [39, 40].

GOG 218 was a double-blind, placebo-controlled, three-arm trial designed to determine whether the incorporation of bevacizumab to standard chemiotherapy (cisplatin and paclitaxel) in first-line treatment improves progression-free survival (PFS) in stage III and IV epithelial ovarian cancer patients who had undergone debulking surgery. The study evaluated bevacizumab added to standard chemotherapy followed by bevacizumab maintenance for 22 cycles (PFS 14.1 months) versus standard chemotherapy (median PFS = 10.3 months). Patients in the third arm received bevacizumab only with chemotherapy and did not have better clinical outcomes than those treated with standard chemotherapy alone (PFS = 11.2 months). Relative to control group, the HR for progression of death was 0.908 (95% CI 0.795–1.040; *p* = 0.16) with bevacizumab-initiation and 0.717 (95% CI 0.625–0.824; *p* < 0.001) with bevacizumab throughout. The lack of a significant difference in PFS between control group and the bevacizumab-initiation group implited that bevacizumab must be continued beyond chemotherapy to delay disease progression [41, 42].

Another trial investigating the efficacy of standard chemotherapy with addition of bevacizumab in patients with OFPC was the ICON7 study (NCT00483782). This phase III randomized, two-arm trial shown that the use of bevacizumab given concurrently with 5 or 6 cycles of platinum-based chemotherapy and continued for an additional 12 cycles improved PFS by about 2 months and increased the response rate by 20%. The PFS and OS benefits were much greater among the patients at high risk for progression [improvements of 3.6 months (restricted mean) and 7.8 months (median) respectively], however, bevacizumab did expand the range of toxic effects such as hyperthension and bowel perforation [43, 44].

Thus, GOG-0218 and ICON7 trials showed that use of bevacizumab maintenance after standard chemotherapy prolongs median PFS in patients with advanced epithelial ovarian cancer [39]. The ongoing phase IV trial MITO16/MANGO-2 (NCT01706120) is intended to explore the potential clinical factors and biological markers identifying patients that will benefit most from addition of bevacizumab to first-line chemotherapy, in terms of progression-free and overall survival. Phase III BOOST trial (NCT01462890) is aimed on evaluation of optimal treatment duration of bevacizumab combination with standard chemotherapy.

Other studies suggest that the patients with recurrent ovarian cancer may benefit from bevacizumab, regardless of the sensitivity to platinum treatment [45]. The first randomized, open-label, phase III trial combining bevacizumab with standard chemotherapy in patients with recurrent platinum-resistant ovarian cancer who were given single-agent chemotherapy alone or with bevacizumab until the disease progression was AURELIA (NCT00976911). Median PFS was 3.4 months with chemotherapy alone versus 6.7 months with bevacizumab-containing therapy (HR = 0.48, 95% CI 0.38–0.60; unstratified log-rank *p* < 0.001). Median OS was 3.3 months longer in the treatment group; however, it was not statistically significant. Safety analysis showed that hypertension and proteinuria were more common in patients treated with chemotherapy and bevacizumab than in the control group. Thus, this study showed that adding bevacizumab to chemotherapy significantly improved PFS and objective response rate (ORR); there was also trend toward longer OS [46].

Another study with final results is OCEANS (NCT00434642), a randomized, placebo-controlled, phase III trial, investigating the efficacy and safety of bevacizumab maintenance after gemcitabine and carboplatin. The patients with platinum-sensitive recurrent ovarian cancer were treated with 6–10 cycles of chemotherapy and then bevacizumab or placebo was continued until disease progression. Median PFS was 4 longer in the treatment group (HR = 0.484; 95% CI 0.388–0.605; log-rank *p* < 0.0001). Median OS was comparable between arms. No new safety signals were identified following prolonged exposure to bevacizumab, however, in experimental group adverse events had greater frequency than in control group [47]. AGO-OVAR17 trial (NCT01462890) is intended to evaluate optimal treatment duration as maintenance.

Three phase III clinical trials of bevacizumab in recurrent ovarian cancer treatment (AURELIA, OCEANS and GOG0213/NCT00565851) that investigated in total 1502 patients, were included into two meta-analyses [48, 49]. Both meta-analyses showed that adding bevacizumab to standard chemotherapy improved ORR, PFS and OS, and it had a higher, but manageable incidence of toxicities (graded 3–4).

It was also tested whether adding bevacizumab to neoadjuvant carboplatin-paclitaxel helps achieve complete resection at IDS, in patients with advanced initially unresectable ovarian cancer (ATHALYA/NCT01739218). Complete resection rate was significantly higher in a group receiving additional bevacizumab. The most common grade 3 adverse reactions to treatment occured in 62% of patients in the bevacizumab group and 63% of patients in the control group. Post-operative complications occurred in 28 and 36% of the patients, respectively [50].

It was observed that bevacizumab can induce macrophage/monocyte infiltration [51] that has been identified as an independent poor prognostic factor in several types of cancer [52]. The major survival factor for these cells is granulocyte–macrophage colony-stimulating factor 1 (GM-CSF1). A trial NCT02923739 is planned that will evaluate efficacy of emactuzumab, an inhibitor of GM-CSF receptor [53], following paclitaxel and bevacizumab, in recurrent platinum-resistant OFPC.

On the other hand, bevacizumab may induce hypoxia in the tumor which may contribute to genomic instability, that is thought to increase the sensitivity of cells to PARP inhibitors [54].

The cost-effectiveness of bevacizumab was analyzed based on the results of ICON7 trial (NCT00483782), which showed that adding bevacizumab (7.5 mg/kg) to standard first-line chemotherapy improves not only PFS but also OS in a pre-specified group of women at high risk of progression (in a post hoc subset analysis of 465 high-risk patients, i.e., stage IIIC with residual disease > 1 cm or stage IV, the OS after standard chemotherapy was 28.8 months compared with 36.6 months in the treatment group; HR = 0.64; 95% CI 0.48–0.85; *p* = 0.002). There were three studies, one conducted from the perspective of the U.S. Medicare system [55], one according to the guidelines of U.K. National Health Service [56] and one for Canadian public health care system [57]. It was estimated that ovarian cancer patients at high risk of progression receiving bevacizumab plus standard chemotherapy experienced a mean incremental quality-adjusted life year (QALY) gain of 0.374 years. The incremental cost-effectiveness ratio (ICER) of bevacizumab was approximately $167,771 per life-year saved (Medicare). In Canadian analysis, the ICER was $95,942 per QALY, while in British study, it was £48,975, which was considered above standard cost-effectiveness threshold (£20,000–£30,000 per QALY) accepted by British National Institute for Health and Care Excellence (NICE).

In conclusion, bevacizumab has been shown to improve PFS for 2–4 months and in some settings also OS, although it is associated with higher degree of side effects. A price reduction would be required for this product to become cost-effective for majority of national health services. So far, there were no predictive biomarkers found that could help to select patients, who could greater benefit from bevacizumab.

### Inhibitors of VEGF receptors

#### Cediranib

Cediranib is anti-angiogenic multikinase inhibitor with activity against all three VEGF receptors (VEGFR1-3). Several phase III trials with cediranib tested against different cancers have produced disappointing results; however, promising activity has been seen with cediranib in ovarian cancer. ICON6 trial (NCT00532194) was a randomized phase III double-blind, placebo-controlled study, which enrolled women with platinum-sensitive relapsed ovarian cancer. It provided the evidence of activity and manageable toxicity of cediranib added to platinum-based chemotherapy and continued as maintenance therapy for up to 18 months. Median PFS of 11.0 months was observed in the group treated with cediranib combined with chemotherapy and then cediranib once-daily maintenance, while 8.7-month PFS was observed in the group receiving placebo during therapy and during maintenance (HR = 0.56; 95% CI 0.44–0.72; *p* < 0.0001). In a group treated with cediranib in combination with chemotherapy then placebo maintenance, median PFS was 9.9 months. Cediranib toxic effect was the most common cause for discontinuation: the most frequent were diarrhea, neutropenia, hypertension, and voice changes [58, 59].

In conclusion, addition of cediranib yielded an improvement in progression-free survival, albeit with added toxic effects.

#### Pazopanib

Pazopanib is a multikinase inhibitor of VEGFR1-3, platelet-derived growth factor receptor α and β (PDGFRA and PDGFRB) and c-Kit. A randomized phase II trial MITO-11 (NCT01644825) was investigating the safety and efficacy of pazopanib in combination with paclitaxel in patients with platinum-resistant ovarian cancer. PFS was significantly longer in the experimental group (median PFS—6.35 versus 3.49 months in placebo group; HR = 0.42; 95% CI 0.25–0.69; *p* = 0.0002). Adverse events included neutropenia, fatigue, leucopenia, hypertension and anemia [60].

A phase III study AGO-OVAR16 (NCT00866697) designed to evaluate the efficacy and safety of pazopanib monotherapy versus placebo in women with OFPC who have not progressed after first-line chemotherapy, has shown better PFS in patients receiving pazopanib (median PFS = 17.9 months) than in placebo group (median PFS = 12.3 months). HR for PFS was 0.77 (*p* = 0.0021). First interim analysis of OS did not suggest any benefit. Grade 3 or 4 adverse events of hypertension (30.8%), neutropenia (9.9%), liver-related toxicity (9.4%), and diarrhea (8.2%) were major side effects [61, 62].

In conclusion, pazopanib shows advantage toward longer PFS, both in the treatment of platinum-resistant/refractory ovarian cancer and in platinum-sensitive maintenance; however, further studies are necessary to identify subgroups of patients in whom improved efficacy may balance toxicity of that treatment.

#### Nintedanib

Nintedanib (BIBF1120) is a next generation, potent triple angiokinase inhibitor of VEGFR1/2/3, FGFR1/2/3 and PDGFRα/β, with lesser activity against RET, Flt-3 and Src. It has demonstrated significant anti-tumor activity in several tumor types in preclinical and clinical studies [63]. In 2014, FDA approved nintedanib for the treatment of idiopatic pulmonary fibrosis. Nintedanib in combination with docetaxel was approved, in 2014, by the European Commission for the treatment of adult patients with locally advanced, metastatic or locally recurrent non-small cell lung cancer [64].

LUME-OVAR 1 (AGO-OVAR 12/NCT01015118) was a randomized, double blind, phase III trial where nintedanib was added to standard first-line chemotherapy, followed by nintedanib maintenance for a maximum of 120 weeks, in patients with advanced epithelial ovarian cancer. This study demonstrated significant improvement in median PFS in the treatment group compared with control group (17.2 versus 16.6 months; HR = 0.84; 95% CI 0.72–0.98; *p* = 0.0239). A more pronounced PFS benefit was observed in a subgroup analysis in patients with < 1 cm residual tumor (21.1 versus 20.8 months; HR = 0.75; 95% CI 0.61–0.92; *p* = 0.005). The most common adverse events were gastrointestinal (diarrhea) and hematological (neutropenia, thrombocytopenia, anemia). Drug-related adverse events leading to death occured in three patients in the nintedanib group and in one patient in the placebo group [65].

A randomized, placebo-controlled, phase II study NCT00710762 checked the maintenance treatment with nintedanib following chemotherapy in patients with resistant or partially platinum-sensitive relapsed ovarian cancer. It has shown a prolongation of PFS at 36 weeks compared with placebo (HR = 0.68; 95% CI 0.44–1.07; *p* = 0.07). There was a higher rate of diarrhea, nausea, vomiting and hepatotoxicity in the nintedanib group [66].

Nintedanib has shorter half-life (7–19 h) than bevacizumab (14–21 days). GINECO-OV119 (CHIVA/NCT01583322) was a randomized, double blind, placebo-controlled phase II study of nintedanib in addition to neoadjuvant chemotherapy and IDS in patients with OFPC. No significant difference was observed between the placebo and the nintedanib group in terms of surgery duration as well as pre-operative and post-operative complications of the IDS [67].

There are currently ongoing phase II trials of nintedanib. METRO-BIBF (NCT01610869) is a randomized, placebo-controlled trial which primary objective is to explore the efficacy and safety of an all oral combination of nintedanib and metronomic cyclophosphamide in patients with multiply-relapsed advanced ovarian cancer, who have completed a minimum of two lines of previous chemotherapy and who for any reason are not suitable for further standard intravenous chemotherapy treatments [68]. Another ongoing phase II trial is NCT01669798 which main purpose is to see if nintedanib can increase the number of women with bevacizumab resistant, persistent, or recurrent epithelial ovarian cancer who do not progress for at least 6 months [69].

Nintedanib in combination with carboplatin and paclitaxel is an active first-line treatment that significantly increases PFS for women with advanced ovarian cancer, but is associated with more gastrointestinal adverse events.

#### Angiopoietin inhibitor

In addition to VEGF, other pathways involved in angiogenesis are also exploited. Angiopoietin 1 and 2 (Ang1/2) bind to Tie-2 receptor, what results in stimulation of endothelial cells proliferation, motility and survival. Trebananib (AMG-386) is a fusion protein that selectively binds Ang1/2, preventing signaling through Tie-2. The available results from two clinical trials: NCT00479817 [70] and TRINOVA-1 (NCT01204749) [71] were included in meta-analysis [49] that showed prolonged PFS (HR = 0.67; 95% CI 0.58–0.77; *p* < 0.00001) and OS (HR = 0.81, 95% CI 0.67–0.99, *p* = 0.04) for trebananib in combination with weekly paclitaxel in women with recurrent, partially platinum-sensitive or -resistant OFPC.

#### Summary of anti-angiogenic therapies

Several anti-angiogenic therapies have been shown effective in improving PFS of recurrent ovarian cancer with a potential benefit of 2–6 months, although with added toxic effects. Anti-angiogenic drugs are given to unselected patients as no predictive markers were found so far. Some data indicate that patients whose tumor blood perfusion or oxygenation increases after the initiation of anti-angiogenic therapy, survive longer than those whose tumor perfusion does not change or decreases [36]. This indicates the directions for further studies. Importantly, when several anti-angiogenic therapies (bevacizumab, VEGFR inhibitors and trebananib) were analyzed in two subgroups of (1) platinum-resistant and (2) platinum-sensitive recurrent ovarian cancer, it was shown that the PFS improved significantly in both groups, while the OS was clearly better in the platinum-sensitive group, but insignificant in the platinum-resistant group [49]. These data suggest that it could be possible to improve survival with a more personalized use of anti-angiogenic agents.

## PARP inhibitors

The Poly(ADP-ribose) Polymerase (PARP) proteins are a family of 17 enzymes involved in a wide range of cellular functions, of which PARP1 and PARP2 are known to be engaged in DNA repair. Cancer treatment with PARP inhibitors (PARPi) exploits the concept of synthetic lethality, a phenomenon in which two genetic mutations are harmless when they occur separately, but can result in cell death when they arise in combination. The first clinical trials which validated the clinical significance of this phenomenon involved the study of PARPi in BRCA1 and BRCA2 (BRCA) mutation carriers with advanced solid tumors. In the BRCA wild-type cells, PARP and BRCA proteins participate in DNA repair via different pathways. In the presence of PARP inhibition, BRCA and other homologous recombination repair pathway proteins carry out error-free DNA repair. In the BRCA-mutated cancer cell, inactivation of both alleles of either BRCA1 or BRCA2 leads to homologous recombination deficiency (HRD). Treating such cells with a PARPi leads to massive DNA damage and cellular lethality (rev. in: [72]). Also, other tumor-specific homologous recombination defects may be potentially exploited, such as somatic BRCA mutations, mutations in ATM, ATR, RAD51, and others [73, 74]. First PARP inhibitor approved for clinical use was olaparib [75].

Multiple trials were designed to evaluate PARP inhibitors in ovarian cancer: (1) in a first-line treatment (SOLO1/NCT01844986, NCT02470585, PRIMA/NCT02655016, PAOLA1/ NCT02477644, NEO/NCT02489006), (2) in the treatment of platinum-sensitive relapse (ENGOT-OV24/AVANOVA/NCT02354131, NCI-OVM1403/NCT02446600, SOLO3/ NCT02282020, ARIEL4/NCT02855944), (3) in maintenance after chemotherapy in platinum-sensitive disease (ENGOT-OV16 NOVA/NCT01847274, SOLO 2/NCT01874353, ARIEL3/NCT01968213) or (4) in the treatment for platinum-resistant disease, and (5) in combination with immune checkpoint inhibitors and other biological drugs (widely reviewed in: [54, 76–78]).

### Olaparib

Olaparib (AZD2281) obtained in 2014 an accelerated approval by FDA for the treatment for advanced ovarian cancer in patients with known or suspected germline BRCA mutation, who have been treated with three or more prior lines of chemotherapy [75]. In the same year, European Medicine Agency (EMA) authorized olaparib as monotherapy in maintenance treatment of patients with platinum sensitive, relapsed BRCA-mutated (germline or somatic) high-grade serous epithelial ovarian cancer who are in complete response (CR) or partial response (PR) following platinum-based chemotherapy.

The approval of olaparib was based on data from Study 19 (AZ19/NCT00753545), a phase II clinical trial that evaluated its efficacy and safety, compared with placebo, in platinum-sensitive relapsed high-grade serous ovarian cancer patients [79]. The study showed that olaparib maintenance therapy significantly prolonged progression-free survival, compared with placebo, in patients with BRCA-mutated ovarian cancer (median PFS 11.2 versus 4.3 months; HR = 0.18; 95% CI 0.10–0.31; *p* < 0.0001). Adverse events related to olaparib were mostly of grade 1 to 2 and included nausea, fatigue, vomiting, taste alteration and anorexia, although grade ≥ 3 adverse events were most frequent in the olaparib group (40%) than in the placebo group (22%). The most pronounced were nausea (2 versus 0%), fatigue (7 versus 3%), anemia (5 versus 1%), neutropenia (4 versus 1%).

In several other trials, olaparib was tested as maintenance therapy alone or in addition to standard chemotherapy, as prior- and post-surgery treatment, and in combination with different new drugs. The SOLO1 study (NCT01844986), conducted in collaboration with the Gynecologic Oncology Group (GOG), was designed to assess the role of maintenance olaparib after frontline chemotherapy for OC patients with germline BRCA mutations. SOLO2, performed in collaboration with the European Network of Gynaecological Oncological Trial (ENGOT) Groups, was investigating the role of maintenance olaparib after two or more lines of chemotherapy for OC patients with germline BRCA mutations. Both trials are randomized, double-blind, placebo-controlled. The primary endpoint was investigator-assessed PFS which median was 19.1 months in the treatment group versus 5.5 months in the placebo group (HR 0.30; 95% CI 0.22–0.41; *p* < 0.0001). The results of blinded independent central review of SOLO2 study were shown in March 2017 at the Society of Gynecologic Oncology Annual Meeting on Women’s Cancer, indicating significantly longer PFS (30.2 months with olaparib versus 5.5 months with placebo; HR = 0.25 (95% 0.18–0.35), *p* < 0.0001) [80]. Based on these data the FDA has granted a priority review to a new application for olaparib as a maintenance therapy in relapsed patients with platinum-sensitive ovarian cancer.

Pooled data from six olaparib trials (two phase I trials and four phase II studies) that recruited women with relapsed disease were used to explore the activity of olaparib in relation to the number of prior treatment lines in patients with germline BRCA*-*mutated ovarian cancer. In the pooled population of 273 patients who had been administered three or more lines of prior chemotherapy, the objective response rate (ORR) was 36% with a 7.4 month median duration [81].

Olaparib is also under investigation in combination with chemotherapy. In a randomized, open-label, phase II study (NCT01081951), patients with platinum-sensitive, recurrent OC received either olaparib with paclitaxel and carboplatin, followed by olaparib maintenance, or paclitaxel and carboplatin without any maintenance treatment. PFS was slightly but significantly improved for the olaparib group versus chemotherapy alone (12.2 versus 9.6 months; HR = 0.51, 95% CI 0.34–0.77; *p* = 0.0012), particularly in patients with BRCA mutations (HR = 0.21, 95% CI 0.08–0.55; *p* = 0.0015) [82]. SOLO3 (NCT02282020) is an ongoing randomized, phase III trial in patients with germline BRCA-mutated, recurrent OC who failed two or more lines of chemotherapy, in which olaparib will be compared with single-agent chemotherapy.

Combined treatment options with a number of other agents are also being assessed. Olaparib was studied in combination with the anti-angiogenic multikinase inhibitor, cediranib. Median PFS was 17.7 months for women treated with cediranib and olaparib (*n* = 44) compared with 9.0 months for those treated with olaparib alone (*n* = 46; HR = 0.42; *p* = 0.005) [81, 83]. OS data were not mature; however, there was a trend toward longer OS in the combination group. Treatment-related adverse effects were more common in patients treated with cediranib plus olaparib than with monotherapy.

Recently, results of phase I studies of olaparib in combination with the PI3K inhibitor BKM120 (NCT01623349) [84] and the AKT inhibitor AZD5363 (NCT02208375) [85] have been reported with evidence of activity in OC.

### Niraparib

Niraparib (MK4827) is an oral, selective PARP-1 and -2 inhibitor that was shown in preclinical studies to induce synthetic lethality in tumors with loss of PTEN and BRCA1 or BRCA2 function [77]. Clinical studies showed that niraparib significantly improved PFS in patients with platinum-sensitive recurrent ovarian cancer, regardless of BRCA mutation or HRD status, although its efficacy was highest in patients with BRCA mutations.

In the end of April 2017, niraparib obtained FDA approval for the maintenance treatment of patients with recurrent OC who are in a CR or PR to platinum-based chemotherapy [86]. Approval was based upon data from the international phase III ENGOT-OV16/NOVA (NCT01847274) trial, a double-blind, placebo-controlled study that enrolled 553 patients. Approximately two thirds of study participants did not have germline BRCA mutations. PFS in a group with germline BRCA mutations was 21.0 months, while in the placebo group—5.5 months (*p* < 0.0001). In the group with non-mutated BRCA but with HRD positive score, PFS was 12.9 months while in placebo group—3.8 months (*p* < 0.0001). Even in a group without mutations and HRD-negative, PFS was longer in niraparib-treated patients (6.0 versus 3.9 months, *p* = 0.02). Niraparib reduced the risk of progression or death by 74% in patients with germline BRCA mutations (HR = 0.26) and by 55% in patients without mutations (HR = 0.45). The most common grade 3/4 adverse reactions to niraparib in the NOVA trial included thrombocytopenia (29%), anemia (25%), neutropenia (20%), and hypertension (9%). The majority of hematologic adverse events were successfully managed via dose modification [87].

The ongoing development program for niraparib includes a Phase III trial in patients who have received first-line treatment for ovarian cancer (PRIMA/NCT02655016) and a registrational Phase II trial in patients who have received multiple lines of treatment for ovarian cancer (QUADRA/NCT02354586). Several combination studies are also underway, including trials of niraparib plus pembrolizumab (TOPACIO/NCT02657889) and niraparib plus bevacizumab (ENGOT-OV24/AVANOVA/NCT02354131).

### Rucaparib

Rucaparib (CO338, AGO14699, PF01367338) is an orally administered, small molecule-based PARP-1, -2 and - 3 inhibitor. In December 2016, the FDA granted an accelerated approval for rucaparib as monotherapy for the treatment of patients with advanced ovarian cancer associated with BRCA mutations (germline and/or somatic) who have been treated with two or more lines of chemotherapy [88, 89].

ARIEL2 (NCT01891344) was a phase II biomarker study that assessed if loss of heterozygosity (LOH) level, can predict response to rucaparib. ARIEL2 enrolled patients with platinum-sensitive, recurrent, high-grade serous or endometrioid ovarian cancer after one or more lines of platinum-based chemotherapy and whose last treatment was platinum-based. The primary objective was to evaluate clinical activity of rucaparib in three subgroups, delineated by BRCA mutation and HRD status (expressed by LOH level, quantified with a next-generation sequencing assay): (1) BRCA-mutated, (2) BRCA-wild type/LOH-high and (3) BRCA-wild type/LOH-low. Median PFS after rucaparib treatment in a group with BRCA mutations was 12.8 months (9.0–14.7), in the LOH high group was 5.7 months (5.3–7.6), and in the LOH low group was 5.2 months (3.6–5.5). PFS was significantly longer in the BRCA mutant and LOH high groups compared with the LOH low group. These results suggested that assessment of tumor LOH can be used to identify patients with BRCA wild-type platinum-sensitive ovarian cancers who might benefit from rucaparib.

The most common grade 3 adverse reactions to rucaparib in the ARIEL2 trial included anemia or low hemoglobin (22%), elevated alanine aminotransferase or aspartate aminotransferase (12%), small intestine obstruction (5%), malignant neoplasm progression (5%) [90, 91].

Rucaparib was also tested as maintenance treatment for platinum-sensitive patients, stratified into three groups in the ARIEL3, a double-blind, placebo-controlled, phase III trial that enrolled 564 women (NCT01968213). PFS after rucaparib treatment in a group with BRCA mutations was 16.6 months (HR = 0.23, *p* < 0.0001), in the HRD-group (including patients with BRCA mutation or BRCA wild type/LOH-high) was 13.6 months (HR = 0.32, *p* < 0.0001), and in the “intent to treat” group (including patients with BRCA mutation, BRCA-wild type/LOH-high, BRCA-wild type/LOH-low and BRCA-wild type/LOH indeterminate) was 10.8 months (HR = 0.37, *p* < 0.0001), while in the placebo group median PFS was 5.4 months. The most common grade 3 or higher adverse reactions to rucaparib in the ARIEL3 trial included anemia (18.8 versus 0.5% in the placebo group) and elevated alanine/aspartate aminotransferase (10.5 versus 0%) [91]. In the ongoing ARIEL4 (NCT02855944) confirmatory study, the primary purpose is to assess the efficacy and safety of rucaparib versus standard chemotherapy in the treatment for relapsed ovarian cancer in patients with BRCA mutation.

### Other PARP inhibitors

There are several other PARPi currently tested for the treatment for different cancers, including ovarian. Veliparib (ABT888) is an orally administered inhibitor of both PARP-1 and - 2 that is extensively studied (currently there are 26 registered trials concerning ovarian cancer), in combination with chemotherapy, and as a single agent (rev in: [77]).

Talazoparib (BMN673) has been very promising in preclinical studies, but it is currently tested mostly in the phase I trials. Phase II trial (NCT 02326844) which tested talazoparib as monotherapy for patients with BRCA-mutated ovarian cancer who had prior PARPi treatment has been terminated.

### Summary of PARP inhibitors

PARPi (olaparib, niraparib) have recently become a standard of care for patients with recurrent BRCA-mutated ovarian cancer. In addition, it was shown that olaparib significantly improved PFS in patients with platinum-sensitive recurrent ovarian cancer, regardless of BRCA mutation. Niraparib showed improved PFS in the same setting, regardless of BRCA mutation and HRD status. It suggests that although efficacy of both agents is highest in BRCA-mutated population, other patients may benefit, too. Other settings are currently extensively tested, e.g., PARPi in primary treatment and in maintenance after primary treatment, PARPi as monotherapy or combined with chemotherapy and/or with other biological agents. However, the utility of PARPi in combination with chemotherapy is concerned with enhanced toxicity, thus more promising are strategies combining PARPi with anti-angiogenic agents, or with inhibitors of the P13K/AKT pathway and new generation of immunotherapy (rev. in: [92]).

As only BRCA1 or BRCA2 mutations and cisplatin sensitivity are accepted predictors of a response to PARPi. Thus, it is now widely accepted that BRCA testing should be offered for all women with ovarian cancer.

Exact characteristics of long-term responders is still to be recognized and a better HRD test is needed [93]. Astra Zeneca AZ HRR test examines mutations in 15 genes related with homologous repair (BRCA1/2, ATM, RAD51B/C/D, RAD54L, FANCJ, FANCL, FANCN, BARD1, CHEK1/2, CDK12, PPP2R2A). However, many of these mutations are of low frequency and some confer only very slight sensitivity to PARPi. Myriad MyChoice test is based on the assessment of three independent indicators of genome instability: telomeric allelic imbalance, large-scale transitions, and LOH. Assay is based on whole genome profiling of single nucleotide polymorphisms (SNPs) [51, 94].

Although current data indicate that PARPi are well tolerated, careful assessment of moderate and late-onset toxicities is required, as these drugs are intended to be taken for a long periods of time.

Clinical studies suggest that PARPi may have a greater impact on prolonging PFS in BRCA-mutated patients then anti-angiogenic therapy. In addition, PARPi are better tolerated, and have a benefit of oral administration. However, cost-effectiveness of this therapy is currently challenging; e.g., according to the appraisal by NICE, the ICER for olaparib maintenance in platinum-sensitive relapsed ovarian cancer versus routine surveillance is likely to be more than £92,000 per QALY gained [90]. It was also suggested by these authors that with limited health care resources, future clinical trials should incorporate a prospective collection of costs, long-term treatment toxicity, and quality of life.

Cost-effectiveness analysis of olaparib and rucaparib was recently presented on American Society of Clinical Oncology 2017 Annual Meeting. Platinum-based combinations were found the most cost-effective at $1672/PFS month, as compared to non-platinum agents ($6688/month), bevacizumab-containing regimens ($12,482/month), olaparib ($13,3731/month), and rucaparib ($14,034/month). Considering a cost of $114,478 for olaparib and $137,068 for rucaparib prior to progression, costs associated with PARPi were 7.1–8.3 times higher than platinum combinations [95, 96]. The authors of this report commented that “while the data on the PARP inhibitors is promising, the unfortunate nature of new therapies is their inherent associated high costs reflecting the high costs of development”.

## EGFR tyrosine kinases inhibitors

ErbB family consists of four closely structurally and functionally related tyrosine kinases: the Epidermal Growth Factor Receptor (EGFR/HER1/ErbB1), Human Epidermal Growth Factor Receptor 2 (HER2/neu/ErbB2), HER3/ErbB3 and HER4/ErbB4. The main ligand of ErbB1 (EGFR) is Epidermal Growth Factor (EGF). ErbB2 has no known ligands, while ErbB3 has no active kinase domain. ErbB receptors may form homodimers or may cooperate by forming heterodimers, both types of interactions resulting in an active signaling trough Ras-Raf-MAPK and PI3K/AKT pathways, what leads to the increased cell proliferation and inhibition of apoptosis. Thus, ErbB proteins are potential therapeutic targets in many cancers.

In ovarian cancer, high EGFR expression was shown to be related with shorter disease-free survival (DFS) and OS. Unfortunately, none of EGFR inhibitors (erlotinib, cetuximab or lapatinib) showed promising results in clinical trials investigating its efficacy in ovarian cancer treatment. Disappointing results were also achieved with pertuzumab which is directed against HER2.

## Folate receptor α inhibitors

Folate receptor alpha (FRα) is glycosylphosphatidylinositol protein, anchored in the cell membrane. In physiological conditions, it is present only in some polarized epithelia and its expression is strictly confined to the apical/luminal cell surface. It is, however, frequently overexpressed in the tumors of epithelial origin, where it loses its polarized location and is present on the entire cell surface. Thus, FRα is a potential biomarker for cancer cells detection and a promising therapeutic target (rev. in: [97]).

Folates play an essential role in the biosynthesis of purines and thymidine, which are required for DNA synthesis, methylation and repair. The majority of folate transport is mediated by low affinity solute transporters, while the proteins from FR family assure high affinity transport. Thus, targeting FRα does not block completely folate intake and this is not a major mechanism responsible for anti-cancer activity of this approach, which is rather related with antibody-dependent cellular cytotoxicity [98].

Ovarian cancer is probably the tumor in which FRα is most frequently overexpressed. It is estimated that over 80% of serous ovarian cancers show FRα expression Lutz [99]. In addition, expression level of FRα has been also correlated with tumor stage and higher histological grade, with poor response to chemotherapy, and worse survival. Moreover, FRα expression is not affected by chemotherapy itself (rev. in: [97, 100]).

FR expression can be exploited therapeutically by several strategies, e.g., using specific antibodies or antibody-like binders to target FRα itself. An example is farletuzumab, which is currently tested in several clinical trials. Other possibility is to use an antibody-drug conjugates to deliver a drug of choice into cancer cell. IMGN853 (mirvetuximab soravtansine) is a representative, currently entering into clinical trials. However, most extensively evaluated are folate–drug conjugates which have been used in several preclinical studies to deliver toxic proteins, radiopharmaceuticals, antisense oligonucleotides, chemotherapeutic agents and their liposomal formulations, to the cancer cells [rev. in: [99]]. A conjugate which is currently tested in clinical trials is vintafolide.

FRα targeting may be also useful for imaging purposes: a small molecule targeting FRα conjugated with technetium-99m-based imaging agent (99mTc-etarfolatide, FolateScan) is tested (NCT03011320) for identification of cells expressing FRα [97].

In addition, anti-FRα vaccines (NCT02111941; NCT02764333) and FRα-targeting T cell therapies are exploited (rev. in: [98, 100]).

### Farletuzumab

Farletuzumab (MORAb-003) is a humanized monoclonal antibody with high affinity for FRα [99]. Preclinical studies suggest that farletuzumab exerts its anti-tumor activity through different mechanisms, either by promotion of tumor cell lysis by antibody-dependent cellular cytotoxicity or complement-dependent cytotoxicity. Other mechanisms are based on induction of sustained autophagy, resulting in a decreased proliferation, or inhibition of the interaction between FRα and lyn kinase, leading to reduced intracellular growth signaling [101]. Phase I/II clinical trials demonstrated feasibility and safety of farletuzumab; the most common adverse events were hypersensitivity reactions, fatigue and diarrhea. A phase II trial (NCT00318370) showed that farletuzumab with carboplatin and taxane may enhance the response rate and duration of response in platinum-sensitive ovarian cancer patients with first relapse after remission of 6–18 months [102]. Unfortunately, the efficacy data in phase III trials are conflicting [103]. One phase III randomized, placebo-controlled trial (NCT00738699) was designed investigate farletuzumab in combination with weekly paclitaxel in patients with platinum-resistant recurrent or refractory EOC. This study was terminated because interim analysis showed that it was unlikely to meet primary endpoint of two-year PFS.

Another phase III randomized, double-blind, placebo-controlled trial (NCT00849667) was aimed to compare the efficacy and safety of six cycles of carboplatin and taxane with and without weekly farletuzumab in patients with a first platinum-sensitive relapse of EOC. No significant differences in PFS among the treatment arms were observed. However, post hoc exploratory analysis revealed a trend toward improved PFS in some patient subsets [101]. It is suggested that lack of improvement in PFS in the above studies was due to the fact that patients were recruited without analyzing FRα expression level. On the other side, usefulness of FRα as a predictive biomarker is unclear. Thus, further studies are necessary to identify biomarkers that will help to define a subgroups of patients that will benefit from this treatment.

### Vintafolide

Vintafolide (MK-8109, EC145) is a water-soluble folate conjugated with microtubule destabilizing agent, a vinca alkaloid derivative, desacetylvin-blastinemonohydrazide (DAVLBH). DAVLBH disrupts the formation of the mitotic spindle, which leads to cell cycle arrest and cell death. Folate–drug conjugate binds to FRα and enters the cell via endocytosis [99]. Early clinical evidence suggested that vintafolide may have anti-tumor effect in women with advanced ovarian cancer [104]. Open-label phase II PRECEDENT trial (NCT00722592) evaluated the effects of adding vintafolide to PLD in patients with platinum-resistant recurrent ovarian cancer. Median PFS was 5.0 months in experimental group versus 2.7 months for PLD alone. The study showed that FRα-positive patients (based on etarfolatide imaging) benefited from vintafolide and PLD combination therapy, whereas patients with FRα-negative tumors did not [103]. Unfortunately, phase III PRECEDENT trial (NCT01170650) has been discontinued because the experimental arm did not meet the pre-specified primary outcome for PFS improvement [105].

### Mirvetuximab soravtansine (IMGN853)

IMGN853 belongs to the class of antibody-drug conjugates; it consists of an anti-FRα antibody coupled to a highly potent cytotoxic maytansinoid payload. It is currently tested in three phase I trials (NCT02996825, NCT01609556, NCT02606305) and one phase III trial (NCT02631876), which is an open-label, randomized study designed to compare the safety and efficacy of IMGN853 to single-agent chemotherapy in women with platinum-resistant FRα-positive advanced EFPC.

## Immunotherapy for ovarian cancer

Cancer immunotherapy includes different approaches aimed to enhance an individual’s own immune system to eliminate tumor cells. EOC is an immunogenic tumor that can be recognized by the host immune system; tumor reactive T cells and antibodies can be detected in the blood, tumor and ascites of EOC patients with advanced disease (rev. in: [106]). It was also shown that higher tumor infiltration with CD8 + T cells (tumor-infiltrating lymphocytes—TILs) is positively correlated with patients survival [107, 108].

Several approaches were designed for either enhancing unspecific immune response or inducing specific adaptive response against tumor antigens, including passive or active immunotherapy (widely reviewed, e.g., in: [109–111]). Unfortunately, although some of the studies reported a positive outcome from the treatment of ovarian cancer with specific immunotherapy, these results were not significant in meta-analysis by Alipour et al. 2016 [112]. More promising seem to be newer approaches involving immune checkpoint inhibitors, alone or in combination with other biological therapies and drugs [110].

### Checkpoint inhibitors and immune modulators

In physiological conditions, distinct immune checkpoint proteins either stimulate or block T lymphocyte activity, to regulate the balance between immune response and tolerance. Checkpoint receptors such as Cytotoxic T Lymphocyte Associated Protein 4 (CTLA-4) and Programmed Cell Death Protein 1 (PD-1) act to reduce autoimmune responses against self-tissues. In cancer patients their activity is often increased, what results in the impaired natural anti-cancer immunity. The rationale behind using immune-checkpoint inhibitors is to unblock anti-tumor responses. Alternatively, activating stimulatory molecules, may be implemented to enhance pre-existing anti-cancer immune responses [109, 113, 114].

Two factors were recognized, so far, that help predict tumor response to immune checkpoint inhibitors, namely accessibility of the tumor by effector immune cells and reliance of tumor cells on immune checkpoint pathways. The surrogate markers for these features are, e.g., the presence of TILs in the tumor and PD-1 ligand (PD-L1) expression, respectively. Based on these markers, it is estimated that over a half of high-grade serous ovarian cancers represents a pattern of adaptive immune resistance and is likely to respond to immune checkpoint inhibitors, while in other histological types such phenotype is less frequent (about 25% of clear cell and mucinous cancers) or absent (low-grade SOC) [Gaillard et al. 2016].

Currently, several immune checkpoint inhibitors are in early phase testing for ovarian cancer treatment (phase I and II) (rev in: [110, 113]).

- Pembrolizumab is an anti-PD-1 antibody, FDA-approved for the treatment for melanoma and NSCLC. Currently, it is tested as monotherapy (NCT02608684, NCT02440425, NCT02537444, Keynote-100/NCT02674061) or in combination with PLD (NCT02865811) or with bevacizumab and cyclophosphamide (NCT02853318) in patients with recurrent ovarian cancer. Pembrolizumab is also evaluated in combination with carboplatin and paclitaxel as a first-line chemotherapy (NCT02520154, NCT02766582);
- Nivolumab is an anti-PD-1 antibody, FDA approved for the treatment for melanoma. It is currently tested in patients with advanced cancers, including OFPC, combined with WT1 analog peptide vaccine plus montanide (an incomplete Freund’s adjuvant), and GM-CSF (a potent stimulator of dendritic cell maturation) (phase I, NCT02737787). Nivolumab is also investigated in combination with oregovomab (anti-CA125 antibody) in phase I/II study (NCT03100006); with bevacizumab (phase II, NCT02873962); or ipilimumab (NCT02498600, NCT02834013, NCT02923934) and in combination with epacadostat (an inhibitor of indoleamine 2,3-dioxygenase; IDO1) (phase I/II, ECHO-204/NCT02327078).
- Ipilimumab is a recombinant, human monoclonal antibody targeting CTLA-4 that is FDA-approved for the treatment for melanoma. It is tested in monotherapy for recurrent platinum-sensitive ovarian cancer (NCT01611558) and in combination with nivolumab (see above).
- Avelumab is a humanized monoclonal anti-PD-L1 antibody that does not block PD-1 interaction with PD-L2. In March 2017, it was FDA approved for the treatment of Merkel cell skin carcinoma. It is currently tested in two Phase III trials for ovarian cancer: one for first-line therapy in combination with carboplatin and paclitaxel (Javelin ovarian 100/NCT02718417) and the other for the treatment for recurrent platinum-resistant/refractory disease, in combination with PLD versus PLD alone (Javelin ovarian 200/NCT02580058)
- Atezolizumab is a humanized, monoclonal antibody targeting PD-L1 that is FDA-approved for the treatment of bladder/urothelial carcinomas. It is tested in several trials for recurrent ovarian cancer, e.g., phase III randomized, double-blinded trial ATALANTE (NCT02891824) that is aimed to evaluate atezolizumab versus placebo in combination with platinum-based chemotherapy and bevacizumab. Phase II randomized trial (EORTC-1508/NCT02659384) is intended to investigate atezolizumab with bevacizumab or acetylsalicylic acid in patients with recurrent platinum-resistant ovarian cancer. Phase II/III randomized study (NCT02839707) is evaluating safety and efficacy of PLD with atezolizumab and/or bevacizumab.
- Durvalumab (MEDI4736) is a monoclonal antibody against PD-L1. It is currently evaluated in phase I/II study (NCT02484404) in combination with olaparib and cediranib in advanced or recurrent ovarian cancer; in phase I/II study (NCT02431559) in combination with PLD and motolimod (a Toll-like receptor 8 agonist), in recurrent platinum-resistant ovarian cancer; in phase I study (NCT01975831) in combinantion with tremelimumab (a human monoclonal antibody against CTLA-4); and in combination with azacitidine (phase I study METADUR/ NCT02811497) in platinum-resistant ovarian cancer. Durvalumab is also tested in combination with TPIV200/huFR-1 (a multi-epitope anti-folate receptor vaccine), in patients with platinum-resistant ovarian cancer (phase II study, NCT02764333). Another phase I/II study (NCT02726997) is aimed to evaluate pharmacodynamics and feasibility of durvalumab in combination with chemotherapy for the first-line treatment of ovarian cancer.

So far, preliminary clinical data show limited efficacy of these agents in ovarian cancer with objective response rates of 10–15% and with some durable responses. Thus, it remains to be established, why some patients do not respond to immune checkpoint inhibitors and to find predictive biomarkers. Another task is to determine the best combination therapy [113].

### Therapeutic vaccines

Therapeutic cancer vaccines are intended to induce cell-mediated immunity, so that immune cells are activated to identify and eliminate malignant cells. For this purpose, selected tumor-associated antigens are delivered using different approaches; there are cell-based vaccines, peptide/protein, epigenetic, and genetic vaccines tested against different tumors, which are either given alone or in combination with different adjuvants, such as cytokines or other stimulatory factors (reviewed in: [106, 115, 116]).

In ovarian cancer, there are several tumor-associated antigen molecules found on the surface or inside the cells that can potentially serve as targets for immune recognition and response; these are, e.g., CA125, p53 protein, FRα, HER2, and cancer–testis antigens, like MAGE-A4 and NY-ESO-1 [117]. Currently, there are mainly pilot and phase I or II trials on the use of therapeutic vaccines in ovarian cancer (widely reviewed in: [106]).

For patients with OFPC there are ongoing studies on p53-MVA vaccine, based on modified vaccinia virus expressing p53 protein (NCT02275039); on an autologous oxidized tumor cell lysate vaccine given with montanide and Poly-ICLC (a Toll-like receptor 3 stimulant) (NCT02452775); on gemogenovatucel-T vaccine that consists of autologous tumor cells electroporated with FANG vector encoding GM-CSF, and a bi-shRNA targeting furin convertase, thereby downregulating endogenous immunosuppressive TGF-β1 and β2 (VITAL/NCT02346747); and on IDO1 inhibitor INCB024360, in combination with CDX-1401 (a fusion protein, consisting of NY-ESO-1 antigen and a human monoclonal antibody against the endocytic dendritic cell receptor, DEC-205) and Poly-ICLC (NCT02166905).

In ovarian cancer, the tumor-specific intra-nodal autologous alpha-DC1 vaccines are tested in phase I/II study NCT02432378. A dendritic cell (DC) vaccine and ontak (denileukin diftitox), a cytotoxic fusion protein containing fragments of diphtheria toxin and human interleukin-2 were tested in already completed study NCT00703105, but no results were published so far. CVac, a MUC1-targeted DC vaccine, was tested in CAN-003/NCT01068509 study. A variable CVac-derived, mucin 1-specific T cell response was measured. PFS was not significantly longer in the treatment group, but one subgroup (patients in second remission) showed an improved PFS and OS [118]. Another study on CVac (NCT01617629) was completed, but no results were published, so far.

Other trials include phase I study (NCT01376505) on a vaccine composed of two HER2 peptides: MVF-HER-2(597–626) and MVF-HER-2 (266–296) tested in different metastatic tumors, including OC; a study on ID-LV305 vaccine, consisting of lentiviral vector targeting DCs, and containing sequences encoding the NY-ESO-1 antigen (NCT02122861); a NCT02387125 trial on CMB305, a combination product composed of a cancer vaccine containing an NY-ESO-1 antigen (LV305) and glucopyranosyl adjuvant in lipid emulsion (G305). A mixed bacteria vaccine (MBV/Coley’s toxin) was tested as non-specific immunotherapy in patients with different tumors expressing NY-ESO-1 antigen in phase I study (NCT00623831). Ten of 12 patients showed a consistent increase in serum IL-6 levels and body temperature. A subgroup of patients showed increasing levels of TNF-α, IFN-γ, and IL1-β [119]. The MVA-5T4 vaccine (a recombinant modified vaccinia Ankara viral vector encoding the 5T4 fetal oncoprotein) is tested in TRIOC/NCT01556841 trial. A phase II/III trial (MIMOSA/NCT00418574) on abagovomab (a murine anti-idiotypic antibody against CA-125) in maintenance therapy was terminated, as no benefit on primary end point (recurrence-free survival) was observed [120].

### Adoptive T cell transfer

A third major trend of immunotherapy for ovarian cancer is adoptive T cell transfer. This therapy uses autologous or allogeneic anti-tumor lymphocytes to induce cancer regression. In this approach, peripheral blood lymphocytes (PBLs) are isolated via apheresis, than tumor-specific lymphocytes are selected and expanded in vitro, then re-introduced into the patient. Alternatively, PBLs can be genetically modified to enhance their anti-tumor activity (rev. in: [109, 121]).

Several phase I and II trials of adoptive T cell transfer are currently underway for patients with advanced cancers, including ovarian, e.g., treatment with NY-ESO-1 antigene-reactive TCR (retroviral vector transduced) autologous PBLs alone (NCT01567891), or with NY-ESO antigene-pulsed dendritic cells as a vaccine (NCT01697527). Other ongoing phase I/II trials are investigating anti-MAGE-A3 antigene-reactive TCR (retroviral transduced) autologous PBLs (NCT02111850) and chimeric antigen receptor (CAR) T cell therapy targeting mesothelin (NCT01583686).

## Palliative treatment for malignant ascites

Advanced and recurrent ovarian cancer is frequently associated with formation of malignant ascites in the peritoneal cavity. Symptoms related with malignant ascites include anorexia, abdominal bloating and pain, dyspnea and respiratory problems, fatigue and insomnia (rev. in: [122]). Mechanisms leading to development of ascites are associated with intraperitoneal spread of tumor cells; current data indicate that the effusion accumulates, e.g., as a result of lymphatic obstruction and increased vascular permeability, mediated by VEGF and interleukin 6 and 8. Malignant ascites may be treated with intraperitoneal administration of radioisotopes or chemotherapy, however, with limited effectiveness. Repetitive paracentesis provides temporary relief of symptoms, but is associated with several side effects, including loss of protein and hypovolemia, circulatory problems and the risk of bowel perforation. The various immunotherapeutic modalities are currently tested for the management of peritoneal metastases and ascites, including T cells, checkpoint inhibitors, antibodies and vaccines (dendritic cell- and virus-based), with promising preclinical results (rev. in: [123]). Recent clinical trials suggest that therapies targeted against VEGF and EpCAM result in slower accumulation of ascites and increase the time to the next paracentesis (rev. in: [122]).

### Catumaxomab

Catumaxomab is a trifunctional rat/mouse hybrid antibody that binds to epithelial cell adhesion molecule (EpCAM) present on tumor cells, to the CD3 antigen on T cells, and to type I, IIa, and III Fcγ receptors on accessory cells (e.g., natural killer cells, dendritic cells, and macrophages). Catumaxomab exerts its anti-tumor effects via T cell-mediated lysis, antibody-dependent, cell-mediated cytotoxicity, and phagocytosis via activation of FcγR-positive accessory cells (rev. in: [124, 125]). In 2009, catumaxomab was approved by EMA for the intraperitoneal treatment of malignant ascites in patients with EpCAM-positive cancer, if a standard therapy is not available. A phase II study (NCT00326885) with IP catumaxomab in platinum-resistant ovarian cancer and recurrent symptomatic malignant ascites showed prolonged time to first therapeutic puncture and puncture-free interval, and a beneficial effect on quality of life, with an acceptable safety profile [126]. In phase II/III trial (EudraCT 2004-000723-15/NCT00836654), puncture-free survival was also significantly longer in the catumaxomab group than in the control group (median 46 versus 11 days; HR = 0.254; *p* < 0.0001) as was median time to next paracentesis (77 versus 13 days; *p* < 0.0001). In addition, catumaxomab patients had fewer signs and ascites associated symptoms than control patients [127].

It was found that patients with soluble EpCAM present in ascites had a significantly shorter overall survival; the prognostic significance was particularly strong in patients with ovarian cancer. However, puncture-free survival and time to next puncture were not significantly different between soluble EpCAM-positive and -negative patients [128].

Phase III study (CASIMAS/NCT00822809) found that IP catumaxomab infusion activates NK cells and macrophages in addition to T cells in ascites and favors CD8(+) T cell accumulation into the peritoneal cavity [129]. In addition, catumaxomab, being a mouse/rat antibody, is able to elicit human anti-mouse antibody (HAMA) reactions. Symptoms can range from a mild allergic reaction, like a rash, to a life-threatening response, such as renal failure. However, in ovarian cancer, it was observed that the elevated HAMA levels were associated with longer median survival, which may indicate a superior anti-tumor immune reactivity in HAMA-positive patients [130, 131].

Catumaxomab was also tested for IV application in patients with EpCAM-positive tumor; however, it was shown in phase I study (NCT01320020) that it caused dose dependent hepatitis. The first patient receiving 10 μg IV catumaxomab experienced fatal acute liver failure which led to the termination of the study [132].

### Aflibercept

Ascites formation is also related with increased vascular permeability caused by VEGF. Aflibercept is a soluble decoy receptor consisting of portions of human VEGF1 and VEGF2 receptors fused to the constant region of human IgG1. It is FDA and EMA approved for the treatment of wet macular degeneration and metastatic colorectal cancer.

A randomized, double-blind, placebo-controlled, phase II trial (NCT00327444) was designed to investigate safety and efficacy of IV aflibercept in inhibition of ascites formation in patients with advanced chemoresistant ovarian cancer. Time to next paracentesis was significantly longer in the experimental group (55.1 days) than in the placebo group (23.3 days). There was no significant difference in overall survival between the experimental and placebo groups [133]. The most frequent adverse events were gastrointestinal disorders, dyspnea, fatigue or asthenia and dehydration. In another phase II study (NCT00396591), median time to next paracentesis was 76.0 days, which was 4.5 times longer than the baseline interval, before aflibercept (16.8 days). Adverse events included hypertension, headache, anorexia, dysphonia, and intestinal perforation (in one patient out of 16 enrolled) [134]. Thus, it seems that aflibercept may be effective for relief the symptoms of malignant ascites, although the major limitation is related with its significant morbidity (risk of bowel perforation) [133, 135].

## Conclusions

Standard treatment for ovarian cancer is surgery, with a goal of complete tumor resection, and chemotherapy based on platinum compounds and taxanes. Definition of “optimal debulking” has changed over years, and now it is clear that survival advantage relies on complete debulking, while leaving any residual tumor, even < 1 cm, is related with worse prognosis. To achieve complete resection, high-quality surgical techniques and sophisticated equipment are required, indicating the need for centralized treatment for ovarian cancer at specialized centers.

Currently, there are many possible new treatment options emerging from recent clinical trials, based both on the modifications of standard approaches and on the addition of a new biological drugs to the standard treatment.

Dose-dense chemotherapy is emerging as an option for patients with poor performance status. The role of IP chemotherapy is still not clear, as well as HIPEC. Both approaches present high level of toxicity/complications and their efficacy has to be confirmed unambiguously in phase III trials.

From among new drugs, bevacizumab and several PARPi were recently approved for ovarian cancer treatment. They are still tested in several settings, including maintenance treatment which is itself an emerging approach with growing applicability and potential.

So far, PARPi show greater efficacy than anti-angiogenic treatment. Unfortunately, finding predictive markers for the response to anti-angiogenic drugs would be very challenging, if possible. Currently, recognized directions in the search for such markers would implicate better understanding the role of tumor microenvironment (hypoxia and perfusion, macrophage infiltration, etc.). Response to PARPi is related with HRD, and several HRD tests are under development. Intriguingly, some patients respond to this therapy despite the lack of HRD signature; this suggests the possibility of finding unknown regulatory circuits of HR and new markers. However, at present, introducing routine BRCA testing for all ovarian cancer patients should be the goal.

Unfortunately, some new drugs appeared unsuccessful, despite strong theoretical rationale behind them, e.g., EGFR inhibitors. Also, folate receptor targeting approaches are not very effective, so far and require further research. New immunotherapeutic approaches based on immune checkpoint inhibitors are currently changing the landscape in melanoma treatment, however, in ovarian cancer potential success of this therapy relies on better understanding of tumor microenvironment and dominant immunosuppressive pathways, as well as finding reliable biomarkers.

Due to its high prevalence, the majority of preclinical and clinical studies concern high-grade serous ovarian cancer. Other, more tailored therapies based on individual histological and biological characteristics should be developed to target less frequent histological types, such as clear cell or low-grade serous carcinoma. According to the specific mutational characteristics of these subtypes, mTOR inhibitors and MEK inhibitors would apply, respectively. Better molecular and genetic characterization of different subtypes of ovarian cancer is required, if we think about personalized treatment.

So far, described biological drugs and new therapeutic approaches were not shown to cure ovarian cancer, but they bring the long awaited promise of turning it into a manageable chronic disease. To bring this promise closer, price reduction of the new drugs is awaited.
